# Do wars abroad affect attitudes at home?

**DOI:** 10.1093/pnasnexus/pgae292

**Published:** 2024-08-20

**Authors:** Margaryta Klymak, Tim Vlandas

**Affiliations:** Department of International Development, Kings College London, London WC2R 2LS, United Kingdom; Department of Social Policy and Intervention, St Antony’s College, University of Oxford, Oxford OX1 2ER, United Kingdom

**Keywords:** attitudes, Russia's invasion, war, external threat, unexpected event during survey design

## Abstract

Can foreign conflicts affect attitudes in nonbelligerent countries? A large literature studies the effects of conflicts and wars on countries that are directly involved, without considering the potential consequences for other nonbelligerent countries that might nevertheless be threatened. To address this question, we examine how the Russian invasion of Ukraine affected 12 economic and political attitudes using survey data covering eight European countries. We use a natural experiment whereby the timing of the invasion overlapped with the fieldwork of a cross-national individual-level survey in these eight countries. We find that the war increased support for democracy, redistribution, support for Europe, and immigration, while it reduced authoritarian attitudes. Our findings highlight the impact of foreign conflicts on a wide range of attitudes in countries that are externally threatened, but neither directly involved militarily, nor necessarily very close to the conflict.

## Introduction

The 2022 Russian invasion of Ukraine has led to over 14.6 million people requiring humanitarian assistance, over 30,000 civilian casualties and 6.5 million Ukrainian refugees (1). Although the invasion has been condemned by national governments and international organizations, it is not clear whether this potential external threat has also affected nonwar-related public attitudes in geographically close, but militarily noninvolved, countries in Europe. We rely on the invasion as a natural experiment to disentangle the effect of experiencing this external threat from the experience of the conflict itself. This brief report provides causal evidence of the impact of a conflict abroad on a wide range of politically relevant attitudes. Our research design leverages the overlap between the timing of the invasion and the fieldwork of the European Social Survey (ESS) (2), where respondents in eight European countries were randomly surveyed just before and right after the invasion. This “Unexpected Event During Survey Design” (UEDSD) method (3, 4) allows us to estimate the causal effects of the invasion on the individual attitudes of respondents in this sample covering eight countries. Since the selection of survey respondents and timing of interviews are fixed in advance, our design resembles a natural experiment: the invasion of Ukraine is a shock to respondents who were interviewed just before compared to just after the start of the war. We find that the invasion has overall affected attitudes in Europe: it reduced authoritarian attitudes whereas it increased support for democracy, Europe, redistribution, and immigration. Thus, conflicts may significantly alter individual attitudes even in nonbelligerent countries.

The full-scale Russian invasion of Ukraine represents a substantial shock to Europe, in terms of the heightened external threat and risk of military spillover, as well as the economic and migration consequences. Our findings contribute to existing literature, reviewed in Section S1.1, that suggests that wars and conflicts have wide-ranging effects on other countries (5–9), most notably the formation of common national and European identity (10–12). Conflicts and wars could also in principle affect nonwarring countries, so this brief report assesses the ramifications of the Russian invasion of Ukraine on a very wide range of attitudes in Europe, which are crucial to understanding Europe's policy response. Whereas governments' fiscal constraints could spur a backlash against providing financial support to refugees, we show that the conflict increased support for redistribution and immigration, consistent with the welcoming stance of governments towards Ukrainian refugees and migrants. In addition, we show that the war led to a “rally around” democratic values and European integration. Finally, we discuss how some of these effects are moderated by country-level refugee inflows and energy reliance, as well as individual ideology.

## Results and discussion

**Fig. 1.**
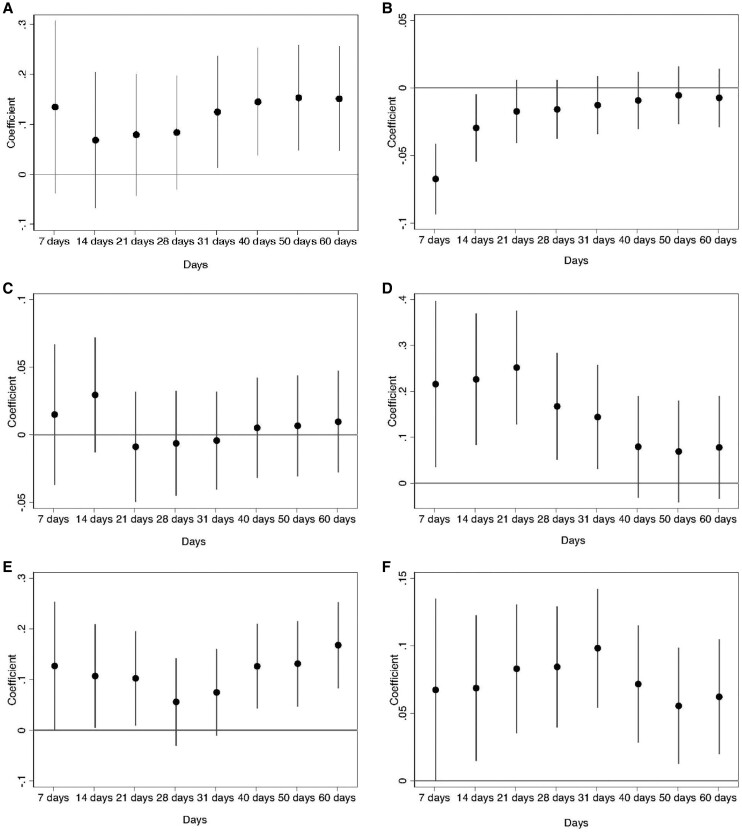
Impact of invasion on attitudes towards Europe, democracy and redistribution. Circles are OLS coefficient estimates (shown on the y-axis) from distinct regressions of each of six dependent variables on a dummy variable taking value one if respondents were interviewed after the start of the invasion, and zero otherwise, for different time bandwidths (shown on the x-axis). The results for each dependent variable are presented in a different panel: A) Emotionally attached to Europe; B) Leave the European Union; C) Join the European Union; D) More EU unification; E) Importance of Democracy; and F) Government should reduce income differences. Horizontal bars are 90% confidence intervals.

**Fig. 2.**
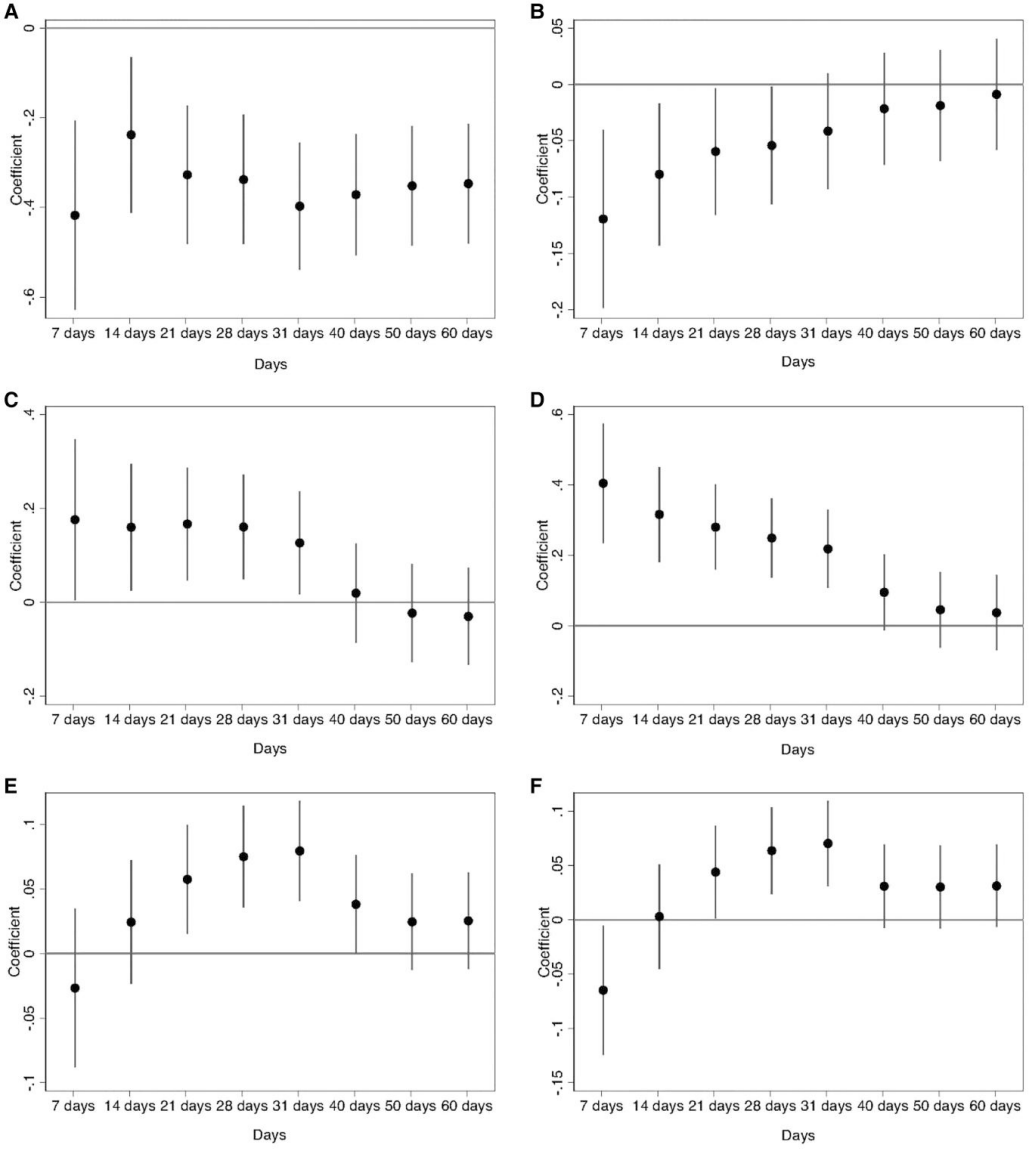
Impact of invasion on attitudes towards authoritarianism and immigration. Circles are OLS coefficient estimates (shown on the y-axis) from distinct regressions of each of six dependent variables on a dummy variable taking value one if respondents were interviewed after the start of the invasion, and zero otherwise, for different time bandwidths (shown on the x-axis). The results for each dependent variable are presented in a different panel: A) Strong leader; B) Loyalty towards the leader; C) Immigration good for the economy; D) Immigration good for culture; E) More immigration from nonmajority group; and F) More immigration from poorer and non-European countries. Horizontal bars are 90% confidence intervals.

The results are shown in Figs 1 and 2. The effect of the invasion on respondents' emotional attachment to the EU only becomes statistically significant after 1 month, where other co-occurring events are less easily ruled out, but there is no effect on joining the EU among nonmember states. The negative effect on support for leaving the EU, and the positive effect on higher support for EU unification, both have statistically significant short to medium-term effects, which fade in the long run. The war also increased the declared importance of living in a democratically governed country and support for government redistribution in both the short and long run. Conversely, respondents were less favorable to having strong leaders above the law and, in the short to medium run, disagreed that a country needs loyalty towards its leaders. Finally, in the first month following the invasion, respondents became more positive about the effect of immigration on their country's economy and culture. As time unfolded, more refugees arrived into Europe, but we find no backlash: on the contrary, respondents became more favorable to welcoming more immigrants into Europe after 1 month. Thus, the effects on some attitudes are immediate, while for others they are only noticeable for larger bandwidths where the effects might become more “bundled” and the mechanisms no longer just about the information of the conflict increasing threat perceptions. We discuss robustness checks in Section S4.

Our analysis of the ESS provides causal evidence that this war abroad significantly affected a range of nonwar-related public attitudes in nonbelligerent European countries that are not directly involved in the conflict. The invasion increased support for democracy, redistribution and Europe, while it reduced authoritarian and anti-immigration attitudes. By contrast, the causal effect of the Iraq and Syrian wars on these European attitudes was not statistically significant (Section S5). Future research should further explore whether these findings generalize to other conflicts, what mechanisms link conflicts and attitudes in nonbelligerent countries, and what other factors may exacerbate these linkages, such as information about the war and the real spill-over effects on other countries.

## Materials and methods

We use the ESS, a cross-national representative individual survey. In eight countries, respondents were surveyed both before and after invasion on 2022 February 24: Switzerland, Greece, Italy, Montenegro, Macedonia, Netherlands, Norway, and Portugal. We estimate the specification:


(1) $$y_{ic}=\alpha +\beta \mathrm{Invasio}n_{i,c}+\gamma X_{i.c}+\gamma_{c}+\epsilon_{i.c}.$$

Our outcome variable, *y_ic_*, captures the attitudes of respondent *i* in a country *c*. Twelve dependent variables are selected based on a literature review in Section S1.2. Our controls **X***_ic_* include the age, gender, residence in urban areas, being foreign born, years of education, subjective income insecurity, and source of income of respondents (see Section S2). Country-specific fixed effects *γ_c_* are included and we report robust standard errors *ɛ_ic_*, but results are robust to other clusters. The effect of the invasion during the ESS fieldwork period is captured by *β*, which represents an exogenous shock to the respondents who were randomly interviewed after the start of the war. We discuss the identification conditions required in UEDSD (3, 4) in Section S3. First, full compliance requires all individuals in the treatment group to be treated with the receipt of information about the start of the war. Second, our treatment is random and not related to respondent characteristics, so individuals of particular age, residence, gender, education or income are not more likely to be treated. Respondents cannot change their interview time, which is set in advance and limits the selective attrition of certain respondents dropping from the sample. We apply entropy rebalancing between treatment and control groups (3) to ensure that the distribution of each covariate is balanced across both groups (Section S2). Third, the exclusion assumption requires ruling out the possibility that unrelated cyclical dynamics, and/or closely occurring events, confound the treatment effect. Tighter bandwidths minimize this risk and unobservable differences between treated and nontreated individuals but also reduce statistical power. We check robustness to the inclusion of a trend and replicate our analyses for different bandwidths: 7, 14, 21, 28, 31, 40, 50, and 60 days after the start of invasion. As the time bandwidth widens, the invasion becomes increasingly more like a “bundled treatment” (Section S3) and other unrelated events may be more likely to drive the observed treatment effect, which complicates the underlying mechanisms. One limitation of the data, which we discuss and explore in more detail in Section S4, is the restricted ability to test treatment effects within each country due to limited statistical power, particularly because two countries (Greece and Italy) have much larger samples. Future research should therefore further explore the timing, geographical heterogeneity and mechanisms of these effects.

## Supplementary Material

pgae292_Supplementary_Data

## Data Availability

The European Social Survey data are freely accessible at https://www.europeansocialsurvey.org/home and both the data and the replication code will be made available on both authors' websites and in a persistent Harvard Dataverse repository (https://dataverse.harvard.edu/) upon acceptance.
