# Supplementary material for: Do wars abroad affect attitudes at home?
Source: PNAS Nexus. 2024 Aug 20;3(8):pgae292. doi: 10.1093/pnasnexus/pgae292 (PMC11333105; doi:10.1093/pnasnexus/pgae292)
Supplement: pgae292_Supplementary_Data [file pgae292_supplementary_data.docx]

**
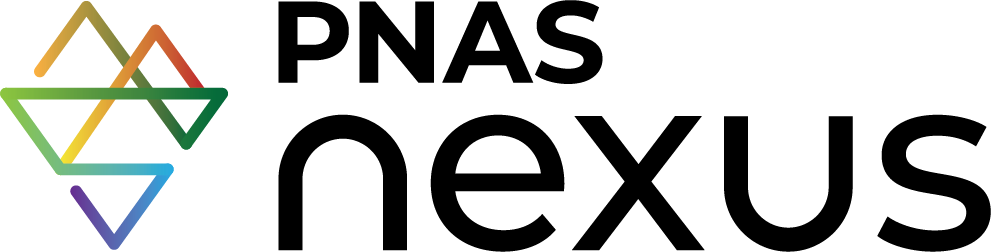
**

**Supplementary Information for:**

Do Wars Abroad Affect Attitudes at Home?

**Authors:**

Margaryta Klymak^a^ and Tim Vlandas^b,^*

^a^ Kings College London, London, UK

^b^ University of Oxford, Oxford, UK

*Corresponding author: [tim.vlandas@spi.ox.ac.uk](mailto:tim.vlandas@spi.ox.ac.uk)

**This PDF file includes:**

Supplementary text

S1. Literature on war and attitudes

S2. More information about data and variables

S3. Identification assumptions

S4. Robustness checks

S5. Extensions and heterogeneity analyses

SI References

NB: no tables or figures are included in this SI consistent with the PNAS Nexus guidelines for brief reports.

**Supporting Information Text**

S1 Literature on war and attitudes

***S1.1. Wars and public opinion in belligerent countries***

There is a large and valuable literature located at the intersection of conflicts, foreign policy, and public opinion. For clarity and simplicity, we categorize existing studies into four separate strands, based on two dimensions, capturing which countries and attitudes are considered. The first dimension differentiates between studies that focus on countries directly involved in a war and those centered on non-belligerent countries. The second dimension distinguishes studies that explore attitudes directly related to foreign policy issues and conflicts from those concerning non-war-related topics. In this brief report, we focus on a fourth scenario, namely the causal impact of war abroad on public opinion at home in non-belligerent countries. We draw on existing theories of what factors shape attitudes to identify four types of attitudes that could *a priori* be affected by wars: attitudes towards Europe, redistribution, democracy and immigration. This brief report aims to estimate the causal effect of the Russian invasion of Ukraine on these attitudes in European neighbor countries.

First, most studies on the intersection of conflicts and attitudes have examined what affects public opinion towards foreign interventions in peace times and towards conflicts in countries that are directly involved in a war (1, 2, 3, 4, 5, 6, 7, 8). For instance, several studies analyzed how and why American public opinion about the Vietnam and Korean wars have evolved over time and varied across individuals (9, 10, 11, 12, 13). Several factors have been identified, most notably the aims behind the decision to participate in the war as well as the perceived legitimacy of the war (14, 6), how political elites frame a conflict (15), the ongoing sacrifices of being at war, both in terms of economic costs and human casualties (16), and the degree and likelihood of success (17). For example, there are important public opinion differences in the UK on the military interventions in Afghanistan and Iraq, where both normative and rational cost-benefit considerations have been shown to shape support for these interventions (8, 18). Another crucial factor influencing support for the war is the type of political regimes. Thus, for instance American and British publics were more supportive of interventions against dictatorships than democracies (19). Foreign policy concerns have in turn been argued to have important implications for electoral outcomes (20), although there is also evidence that the extent of NATO interventions was relatively unaffected by their popularity among the participating countries (21). While valuable in suggesting that the Russian invasion of Ukraine could affect public opinion about the war and foreign policy in these two countries, this literature does not provide us with clear expectations concerning public opinion in non-belligerent countries in Europe that are close to the war.

The second strand of the literature concentrates instead on how wars influence wider public opinion beyond foreign policy attitudes and the war itself, most notably trust in institutions and political actors, satisfaction with government, and other primarily political attitudes. A long lineage of literature has documented that wars and other crises affect several dimensions of domestic public opinion (1, 22). Specifically, national security crises have been linked to ‘rally around the flag’ effects, for example higher support for the incumbent (23, 24), convergence of foreign policy positions between different political parties (25), and greater cooperation (26), although the effect of specific aspects of the war, for instance of casualties, on lower incumbent support is debated (27, 28). This literature therefore points to the possibility that wars and conflict do not only affect foreign policy attitudes and views about the war, but also affect the public trust in their domestic political institutions and actors. However, it has less to say about whether and how this dynamic should be observed in non-belligerent countries, which is what we focus on in this article. This oversight may be important as it underestimates the extent to which wars can affect attitudes not just among belligerent countries but also for those that are not directly involved but might nevertheless be affected in other ways.

Third, fewer studies have explored war-related attitudes in countries that might not be directly involved in a war, but are nevertheless concerned about its impact or indirectly affected. The bulk of these studies have analyzed the unintended negative consequences of US and UK military interventions in the Middle East, such as Iraq, which then led to increased anti-Western attitudes in the Middle East (7, 29, 30). For instance, American interventions in the Middle East since the early 2000s have led to very low favorable views of the US: only 4% of Saudi Arabia, 6% in Jordan and 10% in the United Arab Emirates (31). An analysis of public opinion on the 201 US intervention in Afghanistan across over 60 countries reveals that trade and security alliances with the US as well as religion affected support for the war (32). Interestingly, although NATO membership appeared to increase support for US interventions, other mutual defense pacts seemed to have the opposite effect. There is also some evidence that Western foreign policy interventions have affected public opinion in the Middle East and North Africa (29). Except for Kuwait (33), the conclusion is overall of a ‘backlash’ against Western involvement and to a lesser extent of the political institutions they were officially promoting, i.e. democratic institutions. Global public opinion in turn matters because foreign opinion has a significant effect on the extent of military involvement (34), which is itself linked to public opinion support for attacks on US military targets (35), and also entails second-order effects for other countries (36). Thus, wars can affect public opinion in non-belligerent countries. However, these studies focused on Western democratic countries’ military interventions in mostly non-democratic non-Western countries, whereas our study is concerned with the full-scale invasion of democratic Ukraine by the non-Western illiberal authoritarian Russian state (37). This strand of literature also provides limited guidance about how and why other kinds of attitudes beyond views of democracy and of the West could be affected in our case.

Finally, the fourth quadrant in our two-dimensional categorization of existing literature, which examines non-war related attitudes in countries not directly involved in a conflict, has received minimal attention. Indeed, few studies have concentrated on how inter-state conflicts might affect non-war related attitudes and public opinion in countries that are not directly involved in the conflict, beyond questions about the war itself and the foreign policy of belligerent countries, discussed in previous paragraphs. There are two important exceptions. The first finds that the 2014 Russian invasion of Ukraine led to higher support for Europe among Baltic countries where the perceived military threat was more pronounced (38). While very valuable, this study focused on a much less pronounced security threat for Europe, which therefore could be expected to affect only Eastern European countries, and limited its analysis to attitudes towards Europe. The second article shows that the most recent Russian invasion led to a higher salience in the level of Spanish national identification (39). With this brief report we want to contribute to this emerging literature by concentrating on the more recent Russian invasion of Ukraine and a wider range of attitudes beyond only those towards European integration.

Overall, there is insufficient existing theorization and empirical evidence to date to have clear *ex ante* expectations about whether and how the Russian invasion of Ukraine should affect public opinion in European countries that are not themselves at war. In the next section, we therefore have the more modest ambition to identify which types of attitudes in non-belligerent countries could potentially be expected to change as a result of the non-Western authoritarian Russian state attacking Ukraine, i.e. another country that was in the process of modernizing politically and economically, as well as undergoing a rapprochement with Europe and NATO. We argue that the invasion represented a very substantial and multidimensional shock, which opens up the possibility that many different types of attitudes are affected, not just in Eastern European countries, but also in other countries in Europe, which our research design allows us to include in our analysis.

***S1.2. Russia’s invasion and attitudes in non-belligerent countries***

The Russian invasion of Ukraine represents one of the most significant threat to security and stability of European countries in the last few decades (40). Despite not being directly involved, non-belligerent countries in Europe are therefore directly and indirectly affected in many profound ways. First, the linkages between the Russian invasion of Ukraine and attitudes in Europe could in principle operate via a set of informational channels including information about the war and its consequences from both traditional and social media, declarations from elected politicians in governments as well as from international organizations, for instance the UN and the EU. Inevitably, the information shock is mediated by media and political elites who can shape how the news of the invasion is framed and received by the European public. Although the way that political elites frame the crisis and the declaration they make clearly matters, cues from social peers have also been found to shape foreign policy public opinion (41). Thus, the news of the invasion itself constitutes an information shock that immediately heightened (perceived or real) security threats to people in Europe. In section S3 on identification assumptions, we provide more evidence consistent with compliance operating via higher threats.

Second, another set of less instantaneous channels linking the war to attitudes include several more real-world developments affecting people in Europe. Indeed, in the short to medium run, the Russian invasion also generated substantial negative economic consequences: it destabilized financial markets, disrupted trade and supply chains, and led to exploding inflation, most notably as a result of energy price hikes. Western governments also provided increasing military and economic support to Ukraine (42), including by sanctioning Russia (43). As the war unfolded, the number of migrants and refugees from Ukraine into Europe rose massively. Unlike the direct channel that can be expected to have an effect already in the very short turn, these more indirect channels may take a longer time to fully materialize, hence they can only be captured by longer time bandwidths, which in turn makes it more likely to pick up other unrelated events (see discussion in section S3).

In the next paragraphs, we explain why we expect these direct and indirect effects of the invasion to affect public opinion about democracy, redistribution and immigration attitudes, as well as views about Europe. We selected these attitudes as they directly relate to the nature of the shock (economic and migration) and/or because there is previous literature that has linked increased security threats to these attitudes (European integration and democratic attitudes). That being said, our aim with this brief report is primarily to provide new causal evidence about a phenomenon of immediate impact which we think has the potential to initiate novel avenues of research by providing new facts about contemporary controversies around the political consequences of Russia’s invasion of Ukraine and about long-standing questions on the complex relationship between conflicts and public attitudes.

First, we posit that the Russian invasion of Ukraine increased support for democracy and decreased authoritarian attitudes in Europe. Our starting point for this expectation is the literature mentioned earlier, which has shown that the public in belligerent countries tends to ‘rally around’ their political leaders and institutions (22, 23, 24, 27). Indeed, a number of studies examine how wars between two or more countries influenced public opinion about these countries and their institutions in other non-involved countries. For instance, since the early 2000s, an emerging literature has analyzed the public opinion consequences of Western military interventions in the Middle East, including the first Gulf war, Afghanistan in 2001, and the Iraq War in 2003 (33, 7, 5, 32, 29). These interventions have led to notable changes in evaluations of the US in many countries in the Middle East, and the effectiveness of these military interventions in promoting democratic institutions has been questioned (30). Support for democracy has a result tended to fall as efforts to promote democracy in Iraq became tarnished by the humanitarian, economic and security consequences of protracted warfare. This suggests that neighboring countries turn against not just the aggressor of their neighbor, but also some of the values that they associate with potentially threatening military interventions. In contrast to our case, existing literature has therefore focused on how Western democratic states invading non-Western non-democratic countries affected the attitudes of people living in the Middle East and Muslim countries. Given that the Russian invasion of Ukraine represents nearly the opposite case of a non-democratic non-Western country invading a nascent democratic state that was undergoing a rapprochement with the West and the European Union, we expect the invasion to heighten the (perceived or real) threat that Russia represents, which given its authoritarian nature should have the opposite effect, i.e. lead to a rally around democratic institutions in Europe. This is consistent with the logic underpinning other studies documenting a degree of solidarity between democracies, whereby American and British publics were more supportive of interventions against dictatorships than democracies (19) and people in democratic countries were less likely to support military interventions in other democracies (44). This threat based mechanism operating via an information treatment would in our case lead us to expect a significant positive effect already in the short run, with more mixed expectations in the medium to long run: on the one hand, the initial threat perception could fall as the war does not spill-over into other countries and the Russian invasion stalls, which would lead to a null effect in the medium to long run; on the other hand, the threat perceptions could increase as the conflict enfolds with potentially growing fears of a wider military and nuclear escalation, which would result in the initial short run effect growing larger in the medium to long run.

Second, building on a long lineage of scholarship linking wars and the threat of conflict to the emergence of the welfare state, we have slightly fewer clear-cut expectations about whether redistribution attitudes in Europe to be affected by the Russian invasion of Ukraine. Traditional social policy analyses of the welfare state have consistently argued that wars played a pivotal role in its emergence (45, 46). On the funding side of the welfare state, mass warfare is associated with demands for progressive taxation (47) and the introduction of inheritance taxes (48). In addition, studies have documented a link between wars and the welfare state across fourteen countries between 1860 and 1960 (49), and the Second World War has led to greater social spending (50). There is also evidence concerning the introduction of legislation: both world wars were also associated with the introduction of important welfare legislation in Europe (51). Although the association between conflicts and welfare state expansion is therefore wide ranging and robust, this leaves open the question of what individual level mechanisms (if any) link the two phenomena in our case, so the expectations in this case are more ambiguous. One mechanism linking wars to greater redistribution should in principle only apply to countries that are directly involved: wars tend to lead to destruction of housing and public infrastructure, massive civilian and army casualties, as well as a vast number of army veterans (52). All these negative shocks bring about greater calls for state healthcare services, housing programs and income support schemes. In addition to people involved in wars facing greater risks and insecurities, exposure to violence is also associated with more altruistic behavior (53), while the direct experience of wars increases egalitarian motivations and leads to more pro-redistribution attitudes (54). Previous research has also linked the experience of war to differing perceptions of the importance of luck which in turn reshapes their redistribution attitudes (55, 56). While valuable, these mechanisms all implicitly entailed direct involvement in the conflict for the destruction and violence to influence individual attitudes towards redistribution. Whether the threat of the war spreading to European countries could lead to a greater fear of these negative consequences among the European public, which in turn - in and of itself - would suffice to affect attitudes is an open empirical question.

Moreover, the war can also lead to negative economic and financial shocks in non-belligerent countries, thereby increasing the social risks that the population faces. During war times, uncertainty and risks increase and affect a growing share of the population (57). In turn, higher risks have been linked to the expansion of the welfare state (58). At the individual level, income and property losses are associated with more demands for redistribution (59, 60). At the country level, the development of the welfare state is influenced not only by the war itself but also by related co-occurring phenomena, such as hyperinflation and economic depression, which have been found to spur its growth (61). Because the Russian invasion of Ukraine generated higher perceived or real economic risks and insecurity among the European public, we expect that it is associated with increased support for redistribution. The invasion had substantial negative effects on financial and trade flows, and led to an energy crisis which then fed very high inflation. To the extent that it is not just the direct experience of violence and threats that affect insecurity, but also information about violence and the perception of threat, then this should also reinforce pro-redistribution attitudes. Although threat perceptions can be expected to have an immediate effect on respondents, the effect operating via economic mechanisms could take more time to materialize. If only the perceived threat mechanism effect is at play, we would expect that there is an initial effect that then fades in the medium to longer run, whereas if both mechanisms are present the initial positive effect would increase until it plateaus in the medium to long run. In sum, we test the effect of invasion on redistribution attitudes for completeness, but have fewer clear-cut expectations in this case.

Third, we expect the Russian invasion of Ukraine to affect attitudes towards European integration. Seminal studies of the initial phases of European integration emphasize the crucial importance of the collective memory around World War Two and the motivation to avoid future conflicts on the European continent (62, 63, 64). A long lineage of studies (65, 66, 46) posit that external security threats generated by interstate conflicts were necessary conditions for centralized state building, most notably in the defense and foreign policy domains (67, 68, 69, 70). The path of European integration prior to the Russian invasion of Ukraine, most notably the limited European integration in Defense, is consistent with this logic: the lack of military threat hampered European integration in security policy domains, whereas market pressures led to economic integration (71). This helps make sense of why the EU has developed its legal and economic institutions much more than its ‘coercive’ and security institutions. Several recent contributions have linked threats to individual support for European Defense Policy (72, 73, 74).

In addition, this is the one set of attitudes where there is already some prior evidence linking the earlier 2014 Russian military interventions to support for European integration. Indeed, the 2014 Russian invasion of Ukraine resulted in higher support for Europe among Baltic countries where the perceived military threat was more pronounced (38). We see our brief report as further contributing and adding to these important initial finding by extending the analysis to a more recent, more threatening conflict, using a different identification method (the UESD instead of a difference-in-difference design) as well as considering a wider set of attitudes. Since then, a 2018 survey of the German public (75) further revealed that Russia’s earlier illegal annexation of Crimea in 2014 was associated with higher support for a common European army. Similarly, a survey of 24 EU countries in 2020 (76) showed that perceptions of five different foreign threats, including “Russia’s territorial ambitions”, increased support for EU integration in the security and defense domains. Most recently and consistent with our threat-based mechanisms, a policy brief published shortly before the invasion on the 9th of February 2022 presented survey data from a poll carried out in January 2022 where a majority of respondents saw Russia’s invasion of Ukraine as a threat to European security generally (77).

Finally, the Russian invasion of Ukraine resulted in substantial population flows into Europe. By October 2022, the UNCHR estimated that over 7.5 million Ukrainian refugees were living in Europe and by the end of 2023 there were still around 1 million living in Poland and Germany, respectively. Such a large inflow of people in European countries can be expected to affect immigration attitudes, although there are opposite expectations in the literature about the direction of the effect. On the one hand, the labor market competition thesis posits that natives oppose immigration to avoid competition for jobs in the labor market (78, 79) or because of concerns about the perceived tax implications of providing public services and benefits to refugees (80, 81). Consistent with this logic, refugee and immigration inflows have been linked to support for anti-immigration parties (82, 83) and opposition to immigration is stronger when the economic context deteriorates (84). Given that the substantial flow of Ukrainian refugees coincided with significant economic problems in Europe, we should expect that the Russian invasion led to more anti-immigration attitudes.

On the other hand, more recent evidence highlights instead that non-economic and/or sociotropic consideration may be more important (85). A survey of 18000 voters across 15 European countries for instance demonstrates that attitudes towards hypothetical asylum seekers are more positive when they are Christian rather Muslims, seen as more vulnerable and as more employable, for instance thanks to more skilled previous occupations (86). Women and children also often tend to be perceived as more deserving of public assistance (87, 88). Support is further shaped by the proportionality of refugee allocation across European countries, suggesting that asymmetric allocation might over time undermine public support (89, 90). Closer to our case, De Coninck (91) argues that displaced Ukrainians who have fled to Europe have faced a more positive reception than Afghan refugees because the former were perceived as closer to Europeans and as a result more deserving. Bansak et al (92) report results from conjoint experiments across European countries that show the positive public welcome towards Ukrainian asylum seekers was particularly due to their “demographic, religious and displacement profile”, but this was not “at the expense of support for other marginalized refugee groups, such as Muslim refugees”. Therefore, considering that the majority of Ukrainian refugees comprised vulnerable women and children, who were perceived as more deserving of refuge due to their escape from a war zone caused by the Russian invasion, and given the cultural proximity of Ukrainians to the host countries, the expectations from this literature would be more positive immigration attitudes resulting from the invasion.

Moreover, one could further posit that the effect of the invasion on redistribution attitudes is itself dependent on refugee flows. Indeed, Alesina et al (93) find that redistribution attitudes are negatively correlated with the share of immigration across 140 regions in 16 Western Europe. This would make us expect that the positive effect of the Russian invasion of Ukraine on European redistribution attitudes might disappear beyond a certain level of Ukrainian refugees. This is especially plausible given the very large numbers involved, with over 4.2 million Ukrainians benefited from EU temporary protection up to November 2023 alone (42). But there are also reasons to expect no moderating effect if actual numbers do not relate to perceptions, consistent with a study by Alesina et al (94) showing that people do not correctly estimate immigration flows. To explore this possibility empirically, we have matched the country of ESS respondents to the (log of the) level of refugees in that country. Results (available from the authors) show that the effect of the invasion on positive perceptions of immigration’s impact on a country’s economy and support for redistribution are only statistically significant when the level of refugees is comparatively lower.

In sum, our brief report contributes to the literature by exploring the impact of a major war abroad on attitudes towards non-war related topics in neighboring countries that are indirectly affected and involved, but not directly implicated in the war militarily, facing no direct threats, nor casualties or loss of their infrastructure. Specifically, we analyze how the invasion of a democratic country by a non-Western authoritarian country changes attitudes in Europe. Our review of existing literature helps us identify a range of attitudes in non-belligerent European countries that we could plausibly expect to be affected by a war abroad. Although mechanisms and expectations vary depending on the type of attitudes under consideration, in a nutshell we expect a broad ‘rally effect’ around values associated with the European liberal democratic project, namely democracy and support for Europe, whereas the expectations are more mixed for redistribution and immigration attitudes.

S2 More information about data and variables

We use the ESS, a cross-national individual survey carried out via face-to-face interviews every two years since 2002 in over 20 European countries. Samples are nationally representative while the interview dates are decided at the sampling stage and are not changed. The respondents in eight countries were surveyed both just before and straight after the invasion on 24th February 2022: Switzerland, Greece, Italy, Montenegro, Macedonia, Netherlands, Norway, and Portugal. The frequency of respondents across countries is as follows: 376 (5.20%) in Switzerland; 2,209 (30.54%) in Greece; 2,040 (28.20%) in Italy; 633 (8.75%) in Montenegro; 768 (10.62%) in Macedonia; 510 (7.05%) in Netherlands; 265 (3.66%) in Norway; and 432 (5.97%) in Portugal. In all countries there is a sufficient number of respondents in control and treatment groups (histograms available from authors).

The dependent variables are coded as follows. The first three dependent variables capture attitudes about authoritarianism and democracy. The first dependent variable uses respondents’ answers to the question of whether it is “acceptable for a country to have a strong leader above the law” where they can choose a value on an 11-point scale from 0 “Not at all” to 10 “completely”. The mean answer is 3.16 with a standard deviation equal to 3.15. The second dependent variable uses respondents’ answers to the question whether a “country needs most loyalty towards its leaders” where they can choose a value on a 5-point scale from 1 “Disagree strongly”, 2 “Disagree”, 3 “Neither agree nor disagree”, 4 “Agree”, or 5 “Agree strongly”. The mean answer is 3.22 with a standard deviation equal to 1.12. The third dependent variable uses respondents’ answers to the question of whether they think it is “important for you to live in democratically governed country” where they can choose a value on an 11-point scale from 0 “Not at all important” to 10 ”Extremely important”. The mean answer is 8.83 with a standard deviation equal to 1.85. Next, we include a question about solidarity. Specifically, the fourth dependent variable uses respondents’ answers to the question whether “Government should reduce differences in income levels” where they can choose a value on a 5-point scale from 1 “Disagree strongly”, 2 “Disagree”, 3 “Neither agree nor disagree”, 4 “Agree”, or 5 “Agree strongly”. The mean answer is 1.98 with a standard deviation equal to 0.92.

The next four dependent variables capture attitudes about Europe. The fifth dependent variable uses respondents’ answers to the question of whether they are “emotionally attached to Europe” where they can choose a value on an 11-point scale from 0 “Not at all” to 10 “Very emotionally attached”. The mean answer is 5.51 with a standard deviation equal to 2.59. The sixth dependent variable uses respondents’ answers to the question whether they “would vote for [country] to remain a member of the European Union or to leave the European Union?” where they choose 0 “Remain member of the EU” or 1 “Leave the EU”. This question is only asked to respondents who live in countries that are already members of the European Union. The mean answer is 0.13 with a standard deviation equal to 0.33. The seventh dependent variable uses respondents’ answers to the question whether they “would vote for [country] to become a member of the European Union or to remain outside the European Union?” where they can choose 0 “Remain outside” or 1 ”Become member of EU”. This question is only asked to respondents who do not live in countries that are already members of the European Union. The mean answer is 0.77 with a standard deviation equal to 0.42. The eighth dependent variable uses respondents’ answers to the question of whether they think “European unification go further or gone too far” where they can choose a value on an 11-point scale from 0 “Unification already gone too far” to 10 “Unification go further”. The mean answer is 5.24 with a standard deviation equal to 2.59.

Finally, we include four dependent variables capturing immigration attitudes. The ninth dependent variable uses respondents’ answers to the question of whether “Immigration is bad or good for country’s economy” where they can choose a value on an 11-point scale from 0 “Bad for the economy” to 10 “Good for the economy”. The mean answer is 4.67 with a standard deviation equal to 2.57. The tenth dependent variable uses respondents’ answers to the question whether their “country’s cultural life is undermined or enriched by immigrants where they can choose a value on an 11-point scale from 0 “Cultural life undermined” to 10 ”Cultural life enriched”. The mean answer is 4.91 with a standard deviation equal to 2.62. The eleventh dependent variable uses respondents’ answers to the question whether they support allowing “many/few immigrants of different race/ethnic group from majority come and live here” where they can choose a value on a 4-points scale from 1 “Allow none”, 2 “Allow a few”, 3 “Allow some”, and 4 “Allow many”. The mean answer is 2.67 with a standard deviation equal to 0.97. The twelfth dependent variable uses respondents’ answers to the question whether they support allowing “many/few immigrants from poorer countries outside Europe come and live here” where they can choose a value on a 4-points scale from 1 “Allow none”, 2 “Allow a few”, 3 “Allow some”, and 4 “Allow many”. The mean answer is 2.71 with a standard deviation equal to 0.96.

In terms of controls, we include the following standard controls. For each control, we report the mean and standard deviations for control and treatments, before and after we have applied entropy rebalancing to ensure a covariate balance between treatment and control groups.

- First, we include a variable capturing Education (in years). Pre-entropy balancing, the treatment group mean value is equal to 12.35 (with standard deviation 3.73) compared to a compared to a control group mean equal to 12.31 (with standard deviation 3.68). Post-entropy balancing, the treatment group mean value is equal to 12.35 (with standard deviation 3.73) compared to a compared to a control group mean equal to 12.35 (with standard deviation 3.73).
- Second, we include a variable measuring age (in years). Pre-entropy balancing, the treatment group mean value equal to 50.56 (with standard deviation 18.25) compared to a control group mean equal to 49.57 (with standard deviation 17.76). Post-entropy balancing, the treatment group mean value is equal to 50.06 (with standard deviation 18.25) compared to a control group mean equal to 50.06 (with standard deviation 18.25).
- Third, we include a dummy variable capturing whether the respondent is female. Pre-entropy balancing, the treatment group mean value equal to 0.52 (with standard deviation 0.50) compared to a control group mean equal to 0.53 (with standard deviation 0.50). Post-entropy balancing, the treatment and control group mean values are both equal to 0.52 (both with standard deviation 0.50).
- Fourth, we have a variable measuring income difficulties. Pre-entropy balancing, the treatment group mean value is equal to 2.16 (with standard deviation 0.88) compared to a control group mean equal to 2.13 (with standard deviation 0.87). Post-entropy balancing, the treatment group mean value is equal to 2.16 (with standard deviation 0.88) compared to a control group mean equal to 2.16 (with standard deviation 0.88).
- Fifth, we include a binary variable capturing whether a respondent was born in the country where the interview takes place, and 0 otherwise. Pre-entropy balancing, with treatment group mean value equal to 0.92 (with standard deviation 0.27) compared to a control group mean equal to 0.93 (with standard deviation 0.26). Post-entropy balancing, the treatment group mean value is equal to 0.92 (with standard deviation 0.27) compared to a control group mean equal to 0.92 (with standard deviation 0.27).
- Sixth, we include a dummy variable coded 1 if the respondent is an urban resident, and 0 otherwise. Pre-entropy balancing, with treatment group mean value equal to 0.42 (with standard deviation 0.49) compared to a control group mean equal to 0.41 (with standard deviation 0.49). Post-entropy balancing, the treatment group mean value is equal to 0.42 (with standard deviation 0.49) compared to a control group mean equal to 0.42 (with standard deviation 0.49).

Moreover, we have a series of dummy variable capturing the main source of income where wages are the reference category:

- Main source of income from self-employment. Pre-entropy balancing, the treatment group mean value is equal to 0.11 (with standard deviation 0.32) compared to a control group mean equal to 0. 09 (with standard deviation 0.29). Post-entropy balancing, the treatment group mean value is equal to 0.11 (with standard deviation 0.32) compared to a control group mean equal to 0.11 (with standard deviation 0.32);
- Main source of income from farming. Pre-entropy balancing, the treatment group mean value is equal to 0.02 (with standard deviation 0.15) compared to a control group mean equal to 0.03 (with standard deviation 0.17). Post-entropy balancing, the treatment group mean value is equal to 0.02 (with standard deviation 0.15) compared to a control group mean equal to 0.02 (with standard deviation 0.15);
- Main source of income from pensions. Pre-entropy balancing, the treatment group mean value is equal to 0.26 (with standard deviation 0.44) compared to a control group mean equal to 0.24 (with standard deviation 0.43). Post-entropy balancing, the treatment group mean value is equal to 0.26 (with standard deviation 0.44) compared to a control group mean equal to 0.26 (with standard deviation 0.44);
- Main source of income unemployment benefit. Pre-entropy balancing, the treatment group mean value is equal to 0.01 (with standard deviation 0.08) compared to a control group mean equal to 0.01 (with standard deviation 0.09). Post-entropy balancing, the treatment group mean value is equal to 0.01 (with standard deviation 0.08) compared to a control group mean equal to 0.01 (with standard deviation 0.08);
- Main source of income other benefits. Pre-entropy balancing, the treatment group mean value is equal to 0.02 (with standard deviation 0.13) compared to a control group mean equal to 0.02 (with standard deviation 0.14). Post-entropy balancing, the treatment group mean value is equal to 0.02 (with standard deviation 0.13) compared to a control group mean equal to 0.02 (with standard deviation 0.13);
- Main source of income investments. Pre-entropy balancing, the treatment group mean value is equal to 0.005 (with standard deviation 0.07) compared to a control group mean equal to 0.01 (with standard deviation 0.14). Post-entropy balancing, the group mean value is equal to 0.005 (with standard deviation 0.07) compared to a control group mean equal to 0.005 (with standard deviation 0.07);
- Other income sources. Pre-entropy balancing, the treatment group mean value is equal to 0.03 (with standard deviation 0.18) compared to a control group mean equal to 0.02 (with standard deviation 0.14). Post-entropy balancing, the treatment group mean value is equal to 0.03 (with standard deviation 0.18) compared to a control group mean equal to 0.03 (with standard deviation 0.18).”

S3 Identification assumptions

In this section, we discuss the key conditions for causal identification in ‘Unexpected Event during Survey Design’ (95, 96): full compliance, ignorability and exclusion. First, full compliance requires individuals in the treatment group to have actually been treated. In cases where the treatment is administered by a researcher (e.g. RCT or survey experiments), it is straightforward to assume that all individuals in treated groups have received the treatment. In our case, the treatment is the receipt of information about the start of the war for all respondents to the survey that were interviewed after the invasion has started. It is in general very difficult to formally test this assumption since there is no a priori way to be sure that all respondents were aware that the invasion had started before the time of their interview. That being said, non-compliance is highly unlikely in our case because the Russian invasion of Ukraine was a major event in 2022 that received widespread attention both in print media and online.

Indeed, according to Google’s 2022 report, Ukraine was the third most searched term on their search engine overall and number one in the news category. Compliance was also likely immediate as the invasion occurred before the first interview on February 24th and was widely reported in the morning news across European countries. We find that other surveys are indicative that the war led to heightened threat perceptions in the European public. In a Eurobarometer survey on the war in Ukraine carried out in April 2022 (i.e. a couple of months after the start of the invasion), representative samples of EU citizens were surveyed across the 27 EU member states (97). A necessary condition for news to matter is that people actually paid attention to media during this period: two thirds of respondents followed news several times a day and 20% several times a week, while three quarters discussed it with family and friends weekly. Directly relevant to threat perceptions associated with the invasion, over 80% of respondents declared to be ‘personally worried about the war’ and the perceived risk and fear of escalation was very high: nearly half of respondents feared a ‘third world war’ in Germany, a percentage that is closer to 90% in Ireland, or that it could ‘spread to the rest of Europe’ in Belgium (98). In Italy, 93% were worried about evolution of conflict, while three quarters of Spanish respondents expressed worries about Russia’s use of nuclear weapons (98).

However, beyond the first few days of the Russian invasion, it is also possible that over time the war led to economic and migration consequences that themselves have an impact on people’s attitudes. We treat this shock as a "bundled treatment," following the framework proposed by Enos et al. (99). This implies that the invasion is associated with a combination of different events: the initial attack itself in the short term, the subsequent economic repercussions for Europe, and the policy responses to the invasion in the short to medium term. Western governments responded by providing military and economic support to Ukraine (41) and sanctions were announced against Russia. In April 2022, around 80% of respondents in Europe approved of economic sanctions against Russia, two thirds of financing supply of military equipment to Ukraine, and similarly high support can be observed for EU measures related to energy (100). As a result, we distinguish between two types of mechanisms potentially linking this negative shock to attitudes in Europe: (1) the instantaneous information receipt of the news by respondents, and its effect on the subjective threat perceptions of people; versus (2) a set of non-informational, more ‘real’ mechanisms linking the invasion to certain policy reactions, the economic deterioration, and migration flows into Europe as the war evolved. Note that only the first, informational, mechanism should be observed with very tight time bandwidth, whereas wider bandwidths may also pick up the second, more slow moving, non-informational real effects of the war abroad. Hence as the time bandwidth widens, the invasion becomes increasingly more like a “bundled treatment”.

Second, the ESS ensures in its sampling and weighing protocols that samples are representative and balanced in terms of the characteristics of respondents. Our treatment is random and not related to the distribution of respondent characteristics, so there are in principle no reasons for individuals of particular age, residence, gender, education or income to be more or less likely to have been interviewed after the start of the war. Since respondents cannot change the time of their interview, and since the ESS decided this time in advance, there are no possibilities for certain types of respondents to be more or less likely to pull out after the invasion began. This limits the potential for selective attrition in terms of treated respondents with certain characteristics dropping from the sample. However, it could be that through a purely random allocation process, individuals with certain characteristics end up being over- or under-represented before versus after the start of the war. A balance test shows that there indeed exists a statistically significant difference between the mean values of individuals interviewed after the start of the war, although for very few of the covariates. To address this issue, we apply entropy rebalancing between the treatment and control groups (95). The resulting reweighting effectively ensures that the distribution of each covariate is the same in the reweighted treated and control groups. We report the mean and standard deviations for the control and treatment groups pre- and post-entropy balancing for all control variables in section S2.

Third, the exclusion assumption requires us to exclude the possibility that unrelated cyclical dynamics, and/or other closely occurring events, confound the effect of our treatment. To address this issue, we replicate our analyses for different bandwidths around the start of the war. Reducing the bandwidth entails potential benefits and risks (96). On the upside, reducing the bandwidth makes it more likely that the exclusion restrictions are met. The closer the time of the interview to the date of the treatment cut-off point, the more likely that the treatment is as random as possible for a subset of individuals with relatively fewer unobservable differences in characteristics. This has the additional advantage of limiting the likelihood that other unrelated events drive the observed treatment effect. In terms of risks, a tighter bandwidth reduces the number of individuals included in the sample, while not always ensuring that these individuals share more similar characteristics and will therefore not automatically decrease bias, and at the same time the variance rises. In addition, the smaller sample and shorter time frame limits generalizability by providing more local treatment effects and also rules out more medium term economic and migration channels through which the war could affect attitudes. As a result, if the true treatment effect of the event occurs only over time, a narrower bandwidth might also wrongly suggest a null effect. We therefore use eight different time frames in our analysis: 7, 14, 21, 28, 31, 40, 50 and 60 days after the start of the invasion. As we discuss in section S4, adding a trend does not change our results, and we also carry out placebo tests on different dependent variables where we should not expect an effect, as well as mistiming the timing of invasion.

S4 Robustness checks

We check robustness to changing the sets of controls, including country-fixed effects and a trend, clustering our standard errors at the region-day and country-day levels, restricting our sample only to the five countries with the largest number of observations, jack-knife exclusion of different parts of the sample, reporting sharpened p-values, and to using ordinal logistic models. Specifically, our results are robust to using the following specifications and models (NB: all additional results mentioned in this SI are available from the authors as PNAS nexus does not allow tables nor figures in the SI of a brief report).:

1. Regressing each dependent variable on our treatment without any controls, without weights, and without country fixed effects, while reporting robust standard errors;
2. Regressing each dependent variable on our treatment without any controls, but with weights and country fixed effects, while reporting robust standard errors;
3. Regressing each dependent variable on our treatment while controlling for education, age and gender, including weights and country fixed effects, while reporting robust standard errors;
4. Regressing each dependent variable on our treatment while controlling for education, age, gender and main income source, including weights and country fixed effects, while reporting robust standard errors;
5. Regressing each dependent variable on our treatment while controlling for education, age, gender main income source and proxy for income difficulties, including weights and country fixed effects, while reporting robust standard errors;
6. Regressing each dependent variable on our treatment and all controls (education, age, gender main income source, proxy for income difficulties, urban residence and born in country), including weights and country fixed effects, while reporting standard errors clustered at region-date level;
7. Regressing each dependent variable on our treatment and all controls (education, age, gender main income source, proxy for income difficulties, urban residence and born in country), including weights and country fixed effects, while reporting standard errors clustered at country-date level
8. Ordinal logistic regression with all controls (education, age, gender main income source, proxy for income difficulties, urban residence and born in country), including with weights and country fixed effects, while reporting robust standard errors;
9. Regressing each dependent variable on our treatment and all controls (education, age, gender main income source, proxy for income difficulties, urban residence and born in country), including weights and country fixed effects, while reporting robust standard errors;
10. Regressing each dependent variable on our treatment and all controls (education, age, gender main income source, proxy for income difficulties, urban residence and born in country), including weights, country fixed effects and a trend, while reporting robust standard errors. The statistical significance of our results is not altered when reporting sharpened q-values adjusting for the number of outcomes on which we are running the same specifications [101];
11. Given that certain countries have much smaller samples, and that one must consider not just the distribution of observations across treatment and control groups within each of these countries, but also the distribution of observations and values for the dependent variables of interest, running country specific regressions would risk running into statistical power issues. Thus, one limitation of our data is that the country specific sample size does not allow us to have sufficient power to reliably test the effects in each country separately. Although a jack-knife country exclusion analysis excluding one country at a time suggests that for most dependent variables the effect is statistically significant for at least some bandwidths, this robustness exercise is likely too demanding for this method for three main reasons, and hence we would be reluctant to conclude that some of these results would imply a null finding for a particular outcome. First, such a jack-knife country exclusion is not mentioned nor recommended by the key methodological literature for our method. Second, removing as much as 30% of the total sample in the case of Greece, and to a lesser extent Italy, could lead to some serious statistical power issues. Third, when running multiple regressions for that many outcomes, we could by chance observe null effects even when the true effect is statistically significant. Indeed, with 12 outcomes across 8 countries, and 7 time bandwidths, there are 672 results, hence the probability that they would all be simultaneously statistically significant at the 5% level is extremely low. Thus, we prefer to rely instead on a jack-knife stepwise country-week exclusion, which shows that the distribution of coefficients is stable across all exclusion combinations. The rationale for the country-week jack-knife is to explore whether part of any one country’s sample could be driving the results, without risking the power issues plaguing the country jack-knife. In addition, we conduct explicit hypothesis tests about whether estimates of the treatment effect are significantly different between Greece and Italy on the one hand, and the remaining group of countries on the other hand. We find no obvious evidence that those countries are noticeably different from the rest of the sample for all dependent variables except for three dependent variables capturing whether it is acceptable for a country to have a strong leader above the law, the emotional attachment to Europe, and the government protecting its citizens against poverty, respectively.
12. Finally, we also carry out placebo tests with alternative dependent variables where we should not expect an effect: internet use, trust in scientists, subjective health, a proxy for conspiracy beliefs, religiosity, perception of judicial fairness, the state of education, climate change and safety. The regressions (with weights, country fixed effects, and robust standard errors) suggest that our treatment (Russian invasion of Ukraine) has no statistically significant associations with any of our placebo dependent variables. We further replicate our benchmark analysis to test the effect of the escalation of fights in separatist regions in eastern Ukraine on the dependent variables. The treatment takes the value of zero prior to the 17th of February and one for 14 days after that date. We allow the same duration of the pre-treatment window as in our analysis. We apply entropy balancing for the control group in all regressions and use robust standard errors. As expected we find no statistically significant effect on our dependent variables.

S5 Extensions and Heterogeneity analyses

We carry out several additional empirical tests (NB: all additional results mentioned in this SI are available from the authors as PNAS nexus does not allow tables nor figures in the SI of a brief report). First, we also explored the analyses on two dependent variables measuring individual satisfaction: respondents are asked how satisfied they are with life as a whole on a scale from extremely dissatisfied (coded zero) to extremely satisfied (coded ten); and respondents are asked how happy they are on a scale from extremely unhappy (coded zero) to extremely happy (coded ten). In both cases, the effect of invasion appears negative and statistically significant for most time bandwidths.

Second, although we suspected that the effect of the invasion on European attitudes is linked to the proximity of the conflict as well as the threat that Russia represents, it is arduous to test this intuition without analyzing other conflicts that were further away and where the warring countries were different. Two conflicts did meet the condition of an overlapping timing of invasion with the European Social Survey fieldwork, and represented interesting opposite cases to ours, with a Western democracy invading a non-democratic country that was much farther away from Europe: the US invasions of Iraq on 20th March 2003 and of Syria on 22nd September 2014, which timing overlapped with the survey fieldwork of the first round of the ESS (collected between 01/09/2002 and 15/12/2003) and of the seventh round of the ESS (collected between 01/08/2014 and 13/12/2015), respectively. The estimation strategy and controls are the same as for our baseline analysis, but the country sample inevitably changes as a function of how many countries had ESS survey fieldworks that overlapped with the timing of each invasion and these earlier waves did not have as many of our dependent variables. There was no statistically significant effect of the invasion of Iraq on attitudes in Europe when using the 14 days bandwidths. While we find no effect on views of immigration’s effect on a country’s economy, some statistically significant negative effects on views about immigration’s effect on country’s culture can be observed for bandwidths above 21 days. We further find some evidence for a statistically positive effect on support for redistribution. Finally, we find no effect of invasion of Syria on attitudes in Europe regardless of the bandwidths used.

Third, we match the country of the respondent to the (log of the) level of refugees in that country and re-estimate our model while interacting the invasion dummy with the refugee variable. Our results suggest that the effect of the Russian invasion of Ukraine had a positive effect on perceptions of immigration’s impact on a country’s economy and support for redistribution, but only when the level of refugees is comparatively lower. Fourth, we then test if the invasion’s effects differ depending on country’s reliance on Russian Fossil Fuel Imports. We find that the average marginal effect of the invasion on whether respondents agree that it is important to live in a country that is governed democratically is positive and statistically significant, but not when there is a very high level of energy reliance.

Finally, we explore whether responses differ for left-wing and right-wing respondents. We rely on respondents’ self-placement on a left-right scale (from 0 corresponding to most left-wing to 10 corresponding to the most right-wing), which is not itself significantly affected by the invasion. We then run regressions including an interaction term between this scale and the invasion for three dependent variables of interest: preferences for a stronger leader, which are only negatively affected by the invasion for respondents that are not in the most right-wing self-placement (8, 9 and 10 scores), support for government redistribution, which is only positively affected by the invasion for respondents that are not in the most right-wing self-placement (8, 9 and 10 scores), pro-immigration attitudes, which are only positively affected by the invasion for respondents with center (5, 6 and 7 scores) and right-wing self-placement (8, 9 and 10 scores).

**SI References**

[1] J. E. Mueller, War, presidents, and public opinion. *John Wiley*, 1973.

[2] J. Mueller, Policy and opinion in the Gulf War. *University of Chicago Press*, 1994.

[3] P. Isernia, Z. Juhasz, and H. Rattinger, “Foreign policy and the rational public in comparative perspective,” *Journal of Conflict Resolution*, vol. 46, no. 2, pp. 201–224, 2002.

[4] O. R. Holsti, Public opinion and American foreign policy. *University of Michigan Press*, 2004.

[5] R. C. Eichenberg and R. J. Stoll, “The acceptability of war and support for defense spending: Evidence from fourteen democracies, 2004–2013,” *Journal of Conflict Resolution*, vol. 61, no. 4, pp. 788–813, 2017.

[6] R. C. Eichenberg, “Victory has many friends: Us public opinion and the use of military force, 1981–2005,” *International security*, vol. 30, no. 1, pp. 140–177, 2005.

[7] R. C. Eichenberg, “Global public opinion on the use of military force from the first gulf war to the invasion and occupation of Iraq: Universal logics and national characteristics,” in Presentation to the Convention of the International Studies Association, San Diego, CA, Citeseer, 2006.

[8] J. Reifler, H. D. Clarke, T. J. Scotto, D. Sanders, M. C. Stewart, and P. Whiteley, “Prudence, principle and minimal heuristics: British public opinion toward the use of military force in Afghanistan and libya,” The *British Journal of Politics and International Relations*, vol. 16, no. 1, pp. 28–55, 2014.

[9] S. Verba, R. A. Brody, E. B. Parker, N. H. Nie, N. W. Polsby, P. Ekman, and G. S. Black, “Public opinion and the war in Vietnam,” *American Political Science Review*, vol. 61, no. 2, pp. 317–333, 1967.

[10] E. Schreiber, “Anti-war demonstrations and American public opinion on the war in Vietnam,” *British Journal of Sociology*, pp. 225–236, 1976.

[11] D. C. Hallin, “The media, the war in Vietnam, and political support: A critique of the thesis of an oppositional media,” *Journal of Politics*, vol. 46, no. 1, pp. 2–24, 1984.

[12] S. S. Gartner, G. M. Segura, and M. Wilkening, “All politics are local: Local losses and individual attitudes toward the vietnam war,” *Journal of Conflict Resolution*, vol. 41, no. 5, pp. 669–694, 1997.

[13] R. S. Erikson and L. Stoker, “Caught in the draft: The effects of Vietnam draft lottery status on political attitudes,” *American Political Science Review*, vol. 105, no. 2, pp. 221–237, 2011.

[14] B. W. Jentleson and R. L. Britton, “Still pretty prudent: Post-cold war American public opinion on the use of military force,” *Journal of Conflict Resolution*, vol. 42, no. 4, pp. 395–417, 1998.

[15] A. Gershkoff and S. Kushner, “Shaping public opinion: The 9/11-iraq connection in the bush administration’s rhetoric,” *Perspectives on Politics*, vol. 3, no. 3, pp. 525–537, 2005.

[16] M. T. Koch and S. P. Nicholson, “Death and turnout: The human costs of war and voter participation in democracies,” *American Journal of Political Science*, vol. 60, no. 4, pp. 932–946, 2016.

[17] M. A. Baum and T. Groeling, “Reality asserts itself: Public opinion on Iraq and the elasticity of reality,” *International Organization*, vol. 64, no. 3, pp. 443–479, 2010.

[18] L. A. Hines, R. Gribble, S. Wessely, C. Dandeker, and N. T. Fear, “Are the armed forces understood and supported by the public? a view from the United Kingdom,” *Armed Forces & Society*, vol. 41, no. 4, pp. 688–713, 2015.

[19] R. Johns and G. A. Davies, “Democratic peace or clash of civilizations? target states and support for war in Britain and the united states,” *Journal of Politics*, vol. 74, no. 4, pp. 1038–1052, 2012.

[20] P. A. Klinkner, “Mr. bush’s war: foreign policy in the 2004 election,” *Presidential Studies Quarterly*, vol. 36, no. 2, pp. 281–296, 2006.

[21] S. Kreps, “Elite consensus as a determinant of alliance cohesion: Why public opinion hardly matters for NATO-led operations in Afghanistan,” *Foreign policy analysis*, vol. 6, no. 3, pp. 191–215, 2010.

[22] J. E. Mueller, “Presidential popularity from Truman to Johnson,” *American Political Science Review*, vol. 64, no. 1, pp. 18–34, 1970.

[23] W. D. Baker and J. R. Oneal, “Patriotism or opinion leadership? the nature and origins of the “rally’ round the flag” effect,” *Journal of Conflict Resolution*, vol. 45, no. 5, pp. 661–687, 2001.

[24] J. Hintson and M. Vaishnav, “Who rallies around the flag? nationalist parties, national security, and the 2019 Indian election,” *American Journal of Political Science*, vol. 67, no. 2, pp. 342–357, 2023.

[25] J. M. McCormick and E. R. Wittkopf, “Bipartisanship, partisanship, and ideology in congressional-executive foreign policy relations, 1947-1988,” *Journal of Politics*, vol. 52, no. 4, pp. 1077–1100, 1990.

[26] M. Bauer, C. Blattman, J. Chytilova, J. Henrich, E. Miguel, and T. Mitts, “Can war foster cooperation?,” *Journal of Economic Perspectives*, vol. 30, no. 3, pp. 249–274, 2016.

[27] D. Karol and E. Miguel, “The electoral cost of war: Iraq casualties and the 2004 us presidential election,” *Journal of Politics*, vol. 69, no. 3, pp. 633–648, 2007.

[28] M. T. Koch, “Casualties and incumbents: Do the casualties from interstate conflicts affect incumbent party vote share?,” *British Journal of Political Science*, vol. 41, no. 4, pp. 795–817, 2011.

[29] P. A. Furia and R. E. Lucas, “Determinants of Arab public opinion on foreign relations,” *International Studies Quarterly*, vol. 50, no. 3, pp. 585–605, 2006.

[30] B. B. De Mesquita and G. W. Downs, “Intervention and democracy,” *International Organization*, vol. 60, no. 3, pp. 627–649, 2006.

[31] S. Telhami, “Arab public opinion on the united states and Iraq: Postwar prospects for changing prewar views,” Brookings Review, vol. 21, no. 3, pp. 24–28, 2003.

[32] B. E. Goldsmith, Y. Horiuchi, and T. Inoguchi, “American foreign policy and global opinion: who supported the war in Afghanistan?,” *Journal of Conflict Resolution*, vol. 49, no. 3, pp. 408–429, 2005.

[33] K. Meyer, H. Rizzo, and Y. Ali, “Changed political attitudes in the middle east: The case of Kuwait,” *International sociology*, vol. 22, no. 3, pp. 289–324, 2007.

[34] B. E. Goldsmith and Y. Horiuchi, “In search of soft power: Does foreign public opinion matter for us foreign policy?,” *World Politics*, vol. 64, no. 3, pp. 555–585, 2012.

[35] L. Berger, “Foreign policies or culture: What shapes Muslim public opinion on political violence against the United States?,” *Journal of Peace Research*, vol. 51, no. 6, pp. 782–796, 2014.

[36] S. Ciftci and G. M. Tezcur, “Soft power, religion, and anti-Americanism in the middle east,” *Foreign Policy Analysis*, vol. 12, no. 3, pp. 374–394, 2016.

[37] F. House, “Freedom in the world in 2022. Russia,” 2023.

[38] K. Gehring, “Can external threats foster a European union identity? evidence from Russia’s invasion of Ukraine,” *The Economic Journal*, vol. 132, no. 644, pp. 1489–1516, 2022.

[39] L. Balcells, J. Fernando Tellez, and F. Villamil. 2024. The Wars of Others: The Effect of the Russian Invasion of Ukraine on Spanish Nationalism. *Journal of Politics,* 86(1): 352-357.

[40] NATO, “Vilnius summit communique,” 2023.

[41] J. D. Kertzer and T. Zeitzoff, “A bottom-up theory of public opinion about foreign policy,” *American Journal of Political Science*, vol. 61, no. 3, pp. 543–558, 2017.

[42] C. of the EU and the European Council, “Timeline - EU response to Russia’s invasion of Ukraine,” 2023.

[43] K. Meissner and C. Graziani, “The transformation and design of EU restrictive measures against Russia,” *Journal of European Integration*, vol. 45, no. 3, pp. 377–394, 2023.

[44] M. R. Tomz and J. L. Weeks, “Public opinion and the democratic peace,” *American Political Science Review*, vol. 107, no. 4, pp. 849–865, 2013.

[45] T. H. Marshall et al., Class, citizenship and social development. *Anchor Books*, 1964.

[46] C. Tilly, ed., The Formation of National States in Western Europe. *Princeton University Press*, 1975.

[47] K. Scheve and D. Stasavage, “The conscription of wealth: mass warfare and the demand for progressive taxation,” *International Organization*, vol. 64, no. 4, pp. 529–561, 2010.

[48] K. Scheve and D. Stasavage, “Democracy, war, and wealth: lessons from two centuries of inheritance taxation,” *American Political Science Review*, vol. 106, no. 1, pp. 81–102, 2012.

[49] H. Obinger, K. Petersen, and P. Starke, Warfare and welfare: Military conflict and welfare state development in western countries. *Oxford University Press*, 2018.

[50] H. Obinger and C. Schmitt, “The impact of the second world war on postwar social spending,” *European Journal of Political Research*, vol. 57, no. 2, pp. 496–517, 2018.

[51] H. Obinger and C. Schmitt, “World war and welfare legislation in western countries,” *Journal of European Social Policy*, vol. 30, no. 3, pp. 261–274, 2020.

[52] T. Skocpol, Protecting soldiers and mothers: The political origins of social policy in the United States. Harvard University Press, 1995.

[53] M. J. Voors, E. E. M. Nillesen, P. Verwimp, E. H. Bulte, R. Lensink, and D. P. V. Soest, “Violent conflict and behavior: a field experiment in Burundi,” *American Economic Review*, vol. 102, no. 2, pp. 941–964, 2012.

[54] D. Nettle and R. Saxe, “Preferences for redistribution are sensitive to perceived luck, social homogeneity, war and scarcity,” *Cognition*, vol. 198, p. 104234, 2020.

[55] T. Piketty, Capital in the twenty-first century. *Harvard University Press*, 2014.

[56] A. Alesina and P. Giuliano, “Preferences for redistribution,” in Handbook of social economics, vol. 1, pp. 93–131, Elsevier, 2011.

[57] J. Dryzek and R. E. Goodin, “Risk-sharing and social justice: The motivational foundations of the post-war welfare state,” *British Journal of Political Science*, vol. 16, no. 1, pp. 1–34, 1986.

[58] P. Rehm, Risk inequality and welfare states: Social policy preferences, development, and dynamics. *Cambridge University Press*, 2016.

[59] I. Kuziemko, M. I. Norton, E. Saez, and S. Stantcheva, “How elastic are preferences for redistribution? evidence from randomized survey experiments,” *American Economic Review*, vol. 105, no. 4, pp. 1478–1508, 2015.

[60] G. Gualtieri, M. Nicolini, and F. Sabatini, “Repeated shocks and preferences for redistribution,” *Journal of Economic Behavior & Organization*, vol. 167, pp. 53–71, 2019.

[61] F. G. Castles, “Black swans and elephants on the move: the impact of emergencies on the welfare state,” *Journal of European Social Policy*, vol. 20, no. 2, pp. 91–101, 2010.

[62] E. B. Haas, “The obsolescence of regional integration theory,” 1975.

[63] M. Eilstrup-Sangiovanni and D. Verdier, “European integration as a solution to war,” *European Journal of International Relations*, vol. 11, no. 1, pp. 99–135, 2005.

[64] L. Hooghe and G. Marks, “A postfunctionalist theory of european integration: From permissive consensus to constraining dissensus,” *British Journal of Political Science*, vol. 39, no. 1, pp. 1–23, 2009.

[65] W. H. Riker, “Federalism: Origin, operation, significance,” 1964.

[66] W. H. Riker, “Federalism. handbook of political science, volume 5. F. Greenstein and N. Polsby,” 1975.

[67] S. Skowronek, Building a new American state: The expansion of national administrative capacities, 1877-1920. *Cambridge University Press*, 1982.

[68] M. A. Centeno, Blood and debt: War and the nation-state in Latin America. *Penn State Press*, 2002.

[69] C. G. Thies, “State building, interstate and intrastate rivalry: A study of post-colonial developing country extractive efforts, 1975–2000,” *International Studies Quarterly*, vol. 48, no. 1, pp. 53–72, 2004.

[70] B. D. Taylor and R. Botea, “Tilly tally: War-making and state-making in the contemporary third world,” *International Studies Review*, vol. 10, no. 1, pp. 27–56, 2008.

[71] K. R. McNamara and R. D. Kelemen, “Seeing Europe like a state,” *Journal of European Public Policy*, vol. 29, no. 12, pp. 1916–1927, 2022.

[72] C. J. Carrubba and A. Singh, “A decision theoretic model of public opinion: Guns, butter, and European common defense,” *American journal of political science*, vol. 48, no. 2, pp. 218–231, 2004.

[73] H. Schoen, “Identity, instrumental self-interest and institutional evaluations: Explaining public opinion on common European policies in foreign affairs and defense,” *European Union Politics*, vol. 9, no. 1, pp. 5–29, 2008.

[74] B. Irondelle, F. Merand, and M. Foucault, “Public support for European defense: Does strategic culture matter?,” *European Journal of Political Research*, vol. 54, no. 2, pp. 363–383, 2015.

[75] T. A. Graf, “Unity in the face of threat? exploring the empirical relationship between strategic threat perceptions and public support for a common European army in Germany,” *European Security*, vol. 29, no. 1, pp. 55–73, 2020.

[76] M. Mader and H. Schoen, “No zeitenwende (yet): Early assessment of German public opinion toward foreign and defense policy after Russia’s invasion of Ukraine,” Politische Vierteljahresschrift, pp. 1–23, 2023.

[77] I. Krastev and M. Leonard, “The crisis of European security: What Europeans think about the war in Ukraine,” ECFR Policy Brief. Retrieved from https://ecfr. eu/publication/thecrisis-of-european-security-what-europeans-think-about-the-war-in-ukraine, 2022.

[78] K. F. Scheve and M. J. Slaughter, “Labor market competition and individual preferences over immigration policy,” *Review of Economics and Statistics*, vol. 83, no. 1, pp. 133–145, 2001.

[79] A. M. Mayda, “Who is against immigration? a cross-country investigation of individual attitudes toward immigrants,” *Review of Economics and Statistics*, vol. 88, no. 3, pp. 510–530, 2006.

[80] G. Facchini and A. M. Mayda, “Does the welfare state affect individual attitudes toward immigrants? evidence across countries,” *Review of Economics and Statistics*, vol. 91, no. 2, pp. 295–314, 2009.

[81] G. H. Hanson, K. Scheve, and M. J. Slaughter, “Public finance and individual preferences over globalization strategies,” *Economics & Politics*, vol. 19, no. 1, pp. 1–33, 2007.

[82] A. Steinmayr, “Contact versus exposure: Refugee presence and voting for the far right,” *Review of Economics and Statistics*, vol. 103, no. 2, pp. 310–327, 2021.

[83] E. Dinas, K. Matakos, D. Xefteris, and D. Hangartner, “Waking up the golden dawn: does exposure to the refugee crisis increase support for extreme-right parties?,” *Political Analysis*, vol. 27, no. 2, pp. 244–254, 2019.

[84] R. M. Dancygier and M. J. Donnelly, “Sectoral economies, economic contexts, and attitudes toward immigration,” *Journal of Politics*, vol. 75, no. 1, pp. 17–35, 2013.

[85] J. Hainmueller and M. J. Hiscox, “Attitudes toward highly skilled and low-skilled immigration: Evidence from a survey experiment,” *American Political Science Review*, vol. 104, no. 1, pp. 61–84, 2010.

[86] K. Bansak, J. Hainmueller, and D. Hangartner, “How economic, humanitarian, and religious concerns shape european attitudes toward asylum seekers,” *Science*, vol. 354, no. 6309, pp. 217–222, 2016.

[87] M. Eastmond and H. Ascher, “In the best interest of the child? the politics of vulnerability and negotiations for asylum in sweden,” *Journal of Ethnic and Migration Studies*, vol. 37, no. 8, pp. 1185–1200, 2011.

[88] B. S. Frey, D. A. Savage, and B. Torgler, “Interaction of natural survival instincts and internalized social norms exploring the titanic and lusitania disasters,” *Proceedings of the National Academy of Sciences*, vol. 107, no. 11, pp. 4862–4865, 2010.

[89] K. Bansak, et al., “How economic, humanitarian, and religious concerns shape European attitudes toward asylum seekers”. *Science* **354**,217-222, 2016.

[90] K. Bansak, , Hainmueller, J. & Hangartner, D. “Europeans support a proportional allocation of asylum seekers. *Nat Hum Behav* **1**, 0133, 2017.

[91] D. De Coninck, “The Refugee Paradox During Wartime in Europe: How Ukrainian and Afghan Refugees are (not) Alike”. *International Migration Review*, *57*(2), 578-586, 2023.

[92] K. Bansak, , Hainmueller, J. & Hangartner, D. “Europeans’ support for refugees of varying background is stable over time”. *Nature* **620**, 849–854, 2023.

[93] A. Alesina, Elie Murard, Hillel Rapoport, “Immigration and preferences for redistribution in Europe”, Journal of Economic Geography, **21**, Issue 6, December 2021, Pages 925–954.

[94] A. Alesina, Armando Miano, Stefanie Stantcheva, “Immigration and Redistribution”, The Review of Economic Studies, Volume 90, Issue 1, January, Pages 1–39, 2023.

[95] J. Hainmueller, “Entropy balancing for causal effects: A multivariate reweighting method to produce balanced samples in observational studies,” *Political Analysis*, **20**, no. 1, pp. 25–46, 2012.

[96] J. Munoz, A. Falco-Gimeno, and E. Hernandez, “Unexpected event during survey design: Promise and pitfalls for causal inference,” *Political Analysis*, **28**, no. 2, pp. 186–206, 2020.

[97] Flash Eurobarometer survey 506. Accessed at: <https://europa.eu/eurobarometer/surveys/detail/2772>

[98] European Parliament – “Public opinion on the war in Ukraine”, 2022. Accessed at: <https://www.europarl.europa.eu/at-your-service/files/be-heard/eurobarometer/2022/public-opinion-on-the-war-in-ukraine/en-public-opinion-on-the-war-in-ukraine-20220401.pdf>

[99] R. Enos, Aaron, D., Kaufman, R., and Sands, Melissa L. “Can Violent Protest Change Local Policy Support? Evidence from the Aftermath of the 1992 Los Angeles Riot.” *American Political Science Review* **113,** 4, 1012–28, 2019.

[100] Eurobarometer survey. Accessed at: <https://europa.eu/eurobarometer/surveys/detail/2772>.

[101] M. Anderson “Multiple Inference and Gender Differences in the Effects of Early Intervention: A Reevaluation of the Abecedarian, Perry Preschool, and Early Training Projects”, *Journal of the American Statistical Association*, **103**, 484, 1481-1495, 2008.
